# ASK1 modulates the expression of microRNA Let7A in microglia under high glucose *in vitro* condition

**DOI:** 10.3389/fncel.2015.00198

**Published:** 2015-05-20

**Authors:** Juhyun Song, Jong Eun Lee

**Affiliations:** ^1^Department of Anatomy, Yonsei University College of MedicineSeoul, South Korea; ^2^Brain Korea 21 Plus Project for Medical Sciences, Brain Research Institute, Yonsei University College of MedicineSeoul, South Korea

**Keywords:** Microglia BV2, microRNA-Let7A (miR-Let7A), apoptosis signal regulating kinase 1 (ASK1), cytokine, N-Myc, c-Myc

## Abstract

Hyperglycemia results in oxidative stress and leads to neuronal apoptosis in the brain. Diabetes studies show that microglia participate in the progression of neuropathogenesis through their involvement in inflammation *in vivo* and *in vitro*. In high-glucose-induced inflammation, apoptosis signal regulating kinase 1 (ASK1) triggers the release of apoptosis cytokines and apoptotic gene expression. MicroRNA-Let7A (miR-Let7A) is reported to be a regulator of inflammation. In the present study, we investigated whether miR-Let7A regulates the function of microglia by controlling ASK1 in response to high-glucose-induced oxidative stress. We performed reverse transcription (RT) polymerase chain reaction, Taqman assay, real-time polymerase chain reaction, and immunocytochemistry to confirm the alteration of microglia function. Our results show that miR-Let7A is associated with the activation of ASK1 and the expression of anti-inflammatory cytokine (interleukin (IL)-10) and Mycs (c-Myc and N-Myc). Thus, the relationship between Let-7A and ASK1 could be a novel target for enhancing the beneficial function of microglia in central nervous system (CNS) disorders.

## Introduction

Microglia are major glial cells with an important role in defending the brain against invading microorganisms, as they are the resident immunocompetent and phagocytic cells in the central nervous system (CNS) (Kreutzberg, 1996). However, the improper activation of microglia is related to neuronal damage in CNS diseases via the overproduction of free radicals and the secretion of various cytokines (Dringen, 2005; Krady et al., 2005; Frank-Cannon et al., 2009). Microglia activation is linked to changes in both their morphology, including cell hypertrophy (Tsuda et al., 2008), and their function (Wodarski et al., 2009; Xu et al., 2009). Recently, microglia have been found to exhibit two broad phenotypes: M1 and M2 types (Colton, 2009) Activated M1-type microglia release various neuroinflammatory mediators such as reactive oxygen species (ROS) (Quan et al., 2007) and pro-inflammatory cytokines (Min et al., 2003; Zhang et al., 2013), including interleukin (IL)-1β, IL-6, and tumor necrosis factor (TNF)-α, in the CNS (Hanisch, 2002). By contrast, activated M2-type microglia appear to suppress inflammation by producing anti-inflammatory cytokines, including IL-10 (Yang et al., 2009), tumor growth factor (TGF)-β (Mantovani et al., 2004; Martinez et al., 2008), and IL-4 (Stein et al., 1992). Hyperglycemia impairs neuronal function and leads to oxidative stress (Catanzaro et al., 2013). Cerebral cortex neurons become damaged by high-glucose-induced oxidative stress according to an experimental diabetes study (Arnal et al., 2010). The state of high-glucose induced by hyperglycemia leads to severe neuronal apoptosis in the brain (Knudsen et al., 1989; Sredy et al., 1991; Li and Sima, 2004; Sima et al., 2004) and impaired cognition (Li et al., 2002, 2003). Several studies report that activated microglia are associated with neurodegeneration (Klein et al., 2004) and structural changes, including hippocampal injury and cerebral atrophy, in animal models of diabetes (Musen et al., 2006; Kodl et al., 2008) as well as the progression of brain atrophy and cognitive decline in elderly diabetes patients (van Elderen et al., 2010). Furthermore, a recent *in vitro* study demonstrates that hyperglycemia increases microglial vulnerability to lipopolysaccharide (LPS)-induced inflammation (J. Y. Wang et al., 2001) and induces the secretion of pro-inflammatory cytokines and ROS and the activation of apoptotic signaling pathways, such as the nuclear factor kappa B (NF-κB) signaling pathway (Quan et al., 2007). Some research demonstrates that the inhibition of microglia activation attenuates diabetes-induced inflammatory cytokine production and reduces apoptosis (Krady et al., 2005). How microglia activation is modulated in high-glucose stress is a crucial problem to solve for hyperglycemia-induced neuroinflammatory diseases. Hyperglycemia-induced oxidative stress accelerates cell senescence and activates apoptosis signal regulating kinase 1 (ASK1) (Yokoi et al., 2006), which is a member of the mitogen-activated protein (MAP) kinase kinase kinase group associated with apoptosis signaling (Ichijo et al., 1997; Tobiume et al., 2001). ASK1 can cause apoptosis through non-transcription- or transcription-dependent mechanisms (Tobiume et al., 2001). MicroRNAs (miRNAs), which are endogenous 22-nucleotide RNAs that bind to the 3′-untranslated region of a messenger RNA (mRNA) target, regulate oxidative and inflammatory state (Marin et al., 2013) by affecting gene expression (Martinez and Tuschl, 2004; Du and Zamore, 2005; Schamberger et al., 2012). Several microRNAs including miR-155 (Arango et al., 2015; Cheng et al., 2015; Tan et al., 2015), miR-146 (Schulte et al., 2013; Echavarria et al., 2015), and miR-21(Quinn and O'Neill, 2011) reported the relationship with inflammation in various condition. miR-21, miR-126, and miR-210 are downregulated under *in vitro* diabetic conditions and in Type 1 diabetic patients (Osipova et al., 2014). In addition, miR-146a (Baldeon et al., 2014; Wang et al., 2014), miR-155(Lin et al., 2014), miR-133 (Xiao et al., 2007), miR-1, and miR-206 (Shan et al., 2010) regulate diverse mechanisms in high-glucose stress. Among miRNAs, miR-Let7 in particular is known to regulate glucose metabolism (Zhu et al., 2011) and is linked to inflammatory pathways (Iliopoulos et al., 2009). Here, we investigated the relationship between miR-Let7A and ASK1 activation in microglia function against high-glucose stress. Our results increase our understanding of the mechanisms underlying microglia function in hyperglycemia-induced oxidative stress states.

## Materials and methods

### Microglia culture and D-glucose treatment

Murine BV2 microglial cells were obtained from Prof. Eun-hye Joe (Ajou University School of Medicine, Chronic Inflammatory Disease Research Center) and cultured in Dulbecco's Modified Eagle's Medium (Gibco, NY, USA) supplemented with 10% fetal bovine serum (Gibco, NY, USA) and 100 μg/ml penicillin-streptomycin (Gibco, NY, USA) at 37°C in a humidified atmosphere containing 5% CO_2_. Also we treated D-glucose 25 mM, 60 mM and 90 mM, and 120 mM (Sigma, St. Louis, MO, USA) in microglia cells, considering other *in vitro* studies in high glucose concentrations (25 mM -120 mM) (Quan et al., 2007, 2011; Dey et al., 2015; Zhang et al., 2015).

### miRNA transfection

BV2 microglia were treated with miR-Let7A mimic or anti-miR-Let7A. Let-7A mimic (Let-7A precursor molecules are considered to be processed into mature miRNA by mimicking the Let-7A natural shearing process) and anti-Let-7A (Let7A inhibitor) were purchased from Ambion (Austin, TX, USA). For the transfection of RNA duplexes, a 20 nM final concentration of Let-7A miRNA mimic or Let-7A inhibitor was mixed with lipofectamine 2000 (Invitrogen, Carlsbad, CA, USA) in Opti-MEM medium and incubated at room temperature for 10 min. The mixture was added to cells in 6-well plates. After cells reached 60% confluence, they were harvested for total protein or RNA extraction. ASK1 inhibitor (NQDI-1) was purchased from Tocris Bioscience (Bristol, UK). BV2 microglial cells were pretreated with ASK1 inhibitor (600 nM) to inhibit ASK1 activation 2 h before high-glucose injury.

### Cell viability assay

BV2 microglia (2 × 10^5^ cells/ml) were seeded in 96-well plates to examine the effects of all experimental treatments. Cells were then rinsed with phosphate-buffered saline (PBS), and culture medium was replaced with serum-free medium. Next, 100 μl 3-(4,5-dimethylthiazol-2-yl)-2,5-diphenyltetrazolium bromide (MTT) (Sigma, St. Louis, MO, USA) solution (5 mg/ml in PBS) was added per well. After 1 h of incubation, the medium was removed, and dimethyl sulfoxide was added to solubilize the purple formazan product of the MTT reaction. The supernatant from each well was measured using an ELISA plate reader at a wavelength of 570 nm with background subtraction at 650 nm. All experiments were repeated at least six times. Cell viability was not considered 100% for any treatment group. Cell viability was expressed as a percentage relative to the control group value.

### Reverse transcription polymerase chain reaction (RT-PCR)

To examine the expression of IL-10, c-Myc, and N-Myc in BV2 microglia, RT-PCR was performed using each primer. Briefly, samples were lysed with Trizol reagent (Invitrogen, Carlsbad, CA, USA), and total RNA was extracted according to the manufacturer's protocol. cDNA synthesis from mRNA and sample normalization were performed. PCR was performed using the following thermal cycling conditions: 95°C for 10 min; 35 cycles of denaturing at 95°C for 15 s, annealing at 70°C for 30 s, elongation at 72°C for 30 s; final extension at 72°C for 10 min; and holding at 4°C. PCR was performed using the following primers (5′–3′); IL-10 (F): CCAAGCCTTATCGGAAATGA, (R) TTTTCACAGGGGAGAAATCG; c-Myc (F): TCAAGAGGCGAACACACAAC, (R): GGCCTTTTCATTGTTTTCCA; N-Myc (F): GTCACCACATTCACCATCACTGT, (R): AGCGTGTTCAATTTTCTTTAGCA; GAPDH (F): GGCATGGACTGTGGTCATGAG, (R): TGCACCACCAACTGCTTAGC. PCR products were electrophoresed in 1.5% agarose gels and stained with ethidium bromide.

### Quantitative real-time PCR

To examine the amount of TNF-α, IL-6, IL-10, and ASK1 mRNA in BV2 cells under high-glucose conditions, quantitative real-time PCR was performed using each primer. Total cellular RNA was extracted from BV2 microglia using Trizol reagent (Invitrogen, Carlsbad, CA, USA) according to the manufacturer's instructions. RNA was mixed with One Step SYBR® Prime Script TM RT-PCR Kit II (Takara, Otsu, Shiga, Japan) and specific primers in a total reaction volume of 20 μl. PCR was performed using the following primers (5′–3′); TNF-α (F): CAAGGGACAAGGCTGCCCCG, (R): GCAGGGGCTCTTGACGGCAG; IL-6 (F): AACGATGATGCACTTGCAGA, (R): CTCTGAAGGACTCTGGCTTTG; IL-10 (F): CCAAGCCTTATCGGAAATGA, (R): TTTTCACAGGGGAGAAATCG; ASK1 (F): AGGACGGAGACTGTGAGGGT, (R): GTCCTGCATAGACGATCCCAT; GAPDH (F): GGCATGGACTGTGGTCATGAG, (R): TGCACCACCACTGCTTAGC. Amplification cycles were carried out at 42°C for 5 min, 95°C for 10 s, 95°C for 5 s, 60°C for 34 s, and 65°C for 15 s. Quantitative SYBR Green real-time PCR was performed with an ABI prism 7500 Real-Time PCR System (Life Technologies Corporation, CA, USA) and analyzed with comparative Ct quantification (Popivanova et al., 2008). GAPDH was amplified as an internal control. The Ct value for GAPDH was subtracted from the Ct values for each gene (ΔCt). The ΔCt values of treated cells were compared with those of untreated cells.

### Taqman assay

Total RNA was extracted from cells using Trizol reagent (Invitrogen, Carlsbad, CA, USA) according to the manufacturer's instructions. For quantitative analysis of miR-Let7A, reverse transcription (RT) was performed using Taqman Micro RNA RT kit (Takara, Otsu, Shiga, Japan) according to the manufacturer's instructions with 10 ng total RNA. PCR reactions were then performed according to manufacturer's instructions to quantify the expression level of miR-Let7A using Taqman Universal PCR Master Mix, No Amp Erase UNG (Applied Biosystems, Foster City, CA, USA), and Taqman microRNA assay (Takara, Otsu, Shiga, Japan) for the miR-Let7A of interest. The PCR amplification was performed in ABI 7500 Real Time PCR (Life Technologies Corporation, CA, USA) at 95°C for 10 min, followed by 40 cycles of 95°C for 15 s and 60°C for 60 s. The PCR incubation profile was extended to 40 cycles for miR-Let7A. The PCR primers for Let-7A were (F): 5′-GCGCCTGAGGTAGTAGGTTG-3′ and (R): 5′-CAGTGCAGGGTCCGAGGT-3′. The PCR primers for U6 were (F): 5′-CTCGCTTCGGCAGCACATATACT-3′ and (R): 5′-ACGCTTCACGAATTTGCGTGTC-3′, respectively. The data were uniformly normalized to the internal control U6, and relative expression levels were evaluated using the 2^−ΔΔCt^ method. All experiments were run in triplicate (Zhang et al., 2014).

### Western blot analysis

After drug treatment and exposure to high glucose stress, BV2 cells were washed twice with ice-cold PBS, scraped, and collected. BV2 cell pellets were lysed with ice-cold RIPA buffer (Sigma-Aldrich, St. Louis, MO, USA). The lysates were centrifuged at 13,200 rpm for 1 h at 4°C to produce whole-cell extracts. Protein content was quantified using the BCA protein assay kit (Pierce, IL, USA). Protein (30 μg) was separated on a 10% SDS-polyacrylamide gel and transferred onto a polyvinylidene difluoride membrane. After blocking with 5% bovine serum albumin in TBS/Tween (20 nM Tris (pH 7.2), 150 mM NaCl, 0.1% Tween 20) for 1 h at room temperature, immunoblots were incubated overnight at 4°C with primary antibodies that specifically detect phosphorylation-ASK1 (p-ASK1) (1:1000; Cell signaling biotechnology, Danvers, MA, USA), ASK1 (1:1000; Santa Cruz Biotechnology, Santa Cruz, CA, USA), or β-actin (1:2000; Cell Signaling, Danvers, MA, USA). Blots were then incubated with horseradish peroxidase-linked anti-mouse or -rabbit IgG antibodies (Abcam, Cambridge, MA, USA) for 1 h at room temperature. Protein bands were detected and analyzed using enhanced chemiluminescence (ECL; Pierce, IL, USA)(Song et al., 2014).

### Immunocytochemistry

BV2 microglia were washed three times with PBS for immunostaining and blocked for 30 min. BV2 microglia were then incubated with primary antibodies overnight at 4°C. The following primary antibodies were used: anti-goat CD68 (1:200, Millipore, Billerica, MA, USA) and anti-rabbit CD206 (1:200, Millipore, Billerica, MA, USA). After incubating BV2 microglia with the primary antibodies, cells were washed with PBS three times for 3 min each. Next, samples were incubated with fluorescein isothiocyanate (FITC)-conjugated donkey anti-goat (1:200, Jackson Immunoresearch, West Grove, PA, USA) or rhodamine-conjugated goat anti-rabbit (1:200, Jackson Immunoresearch, West Grove, PA, USA) for 2 h at room temperature. BV2 microglia were washed with PBS three times for 3 min each and then counterstained with 1 μg/ml 4′, 6-diamidino-2-phenylindole (DAPI) (1:100, Invitrogen, Carlsbad, CA, USA) for 10 min at room temperature. Fixed samples were imaged using a Zeiss LSM 700 confocal microscope (Carl Zeiss, Thornwood, NY, USA).

### Statistical analysis

Statistical analyses were carried out using SPSS 18.0 software (IBM Corp., Armonk, NY, USA). All data are expressed as mean ± standard error of the mean (S.E.M.). Significant group differences were determined by one-way analysis of variance followed by Bonferroni *post-hoc* multiple comparisons tests. Each experiment consisted of three replicates per condition. Differences were considered statistically significant at ^*^p < 0.05 and ^**^p < 0.001.

## Results

### Inhibition of ASK1 activation enhances cell viability against high-glucose stress

**Figure 1:**
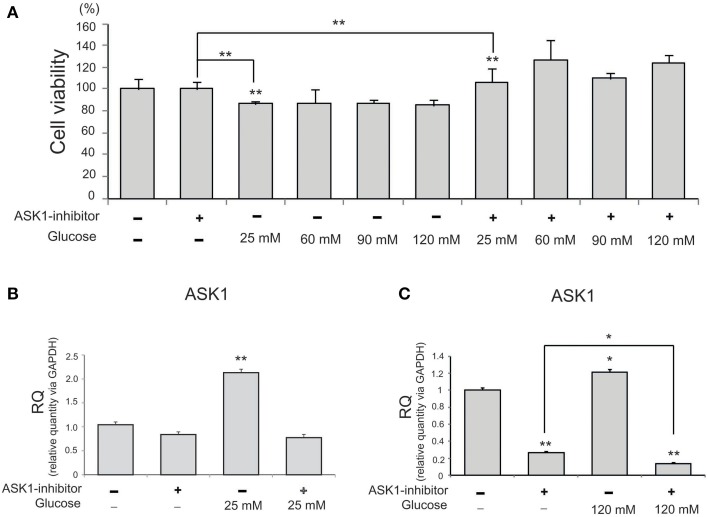
**The measurement of cell viability and the expression of ASK1 in microglia under high glucose condition. (A)** MTT assay. The graph showed that BV2 microglia in the high glucose treatment groups (25, 60, 90, 120 mM) exhibited decrease of cell viability compared to the normal control group. BV2 microglia in all ASK1 inhibitor pre-treatment groups exhibited increase of cell viability after high glucose injury compared to the normal control group. Especially, the microglia in the glucose 120 mM with ASK1 inhibitor conditions showed markedly the increase of cell viability comparison with ASK1 inhibitor treatment group. Each experiment included the six repeats per condition. **(B)** The measurement of relative quantitative mRNA level. The graph showed the mRNA level of ASK1 in glucose condition. In high glucose 25 mM treatment group, the level of ASK1 mRNA increased evidently compared to the no treatment group. ASK1 inhibitor pre-treatment attenuated the expression of ASK1 in microglia in spite of 25 mM high glucose condition. GAPDH was used as a control. Each experiment included the three repeats per condition. Each experiment included the three repeats per condition. Data were expressed as mean ± S.E.M, and were analyzed statistically using One-Way ANOVA, followed by Bonferroni's *post hoc*. ^**^p < 0.001. **(C)** The measurement of relative quantitative mRNA level. The graph showed the mRNA level of ASK1 in glucose condition. In high glucose 120 mM treatment group, the level of ASK1 mRNA increased evidently compared to the no treatment group. ASK1 inhibitor pre-treatment attenuated the expression of ASK1 in microglia in spite of 120 mM high glucose condition. GAPDH was used as a control. Each experiment included the three repeats per condition. Each experiment included the three repeats per condition. Data were expressed as mean ± S.E.M, and were analyzed statistically using One-Way ANOVA, followed by Bonferroni's *post hoc*. ^*^p < 0.05, ^**^p < 0.001. RQ, relative quantitative value of mRNA via GAPDH.

To investigate the viability of BV2 microglia in high-glucose conditions, we conducted an MTT assay with different concentration of glucose (Figure 1A). Cell viability was reduced by glucose treatment (25, 60, 90, or 120 mM). After pre-treatment with ASK1 inhibitor (600 nM, TOCRIS, Bristol, UK), the viability of high-glucose-exposed microglia significantly increased by 20% compared with that of the high-glucose treatment alone group (Figure 1A). Because cell viability showed the largest change by ASK1 inactivation in the 120 mM glucose condition, we used this concentration in subsequent experiments. To determine the whether ASK1 directly participates in the alteration of cell viability against high-glucose stress, we conducted quantitative real time-PCR using ASK1-specific primer (Figures 1B,C). ASK1 expression was increased in the 25 mM glucose condition and was markedly reduced after ASK1 inhibitor pre-treatment (Figure 1B). ASK1 expression was increased in the 120 mM glucose condition and was significantly reduced after ASK1 inhibitor pre-treatment (Figure 1C).

### Measurement of M1- and M2-type microglia after high-glucose injury

**Figure 2:**
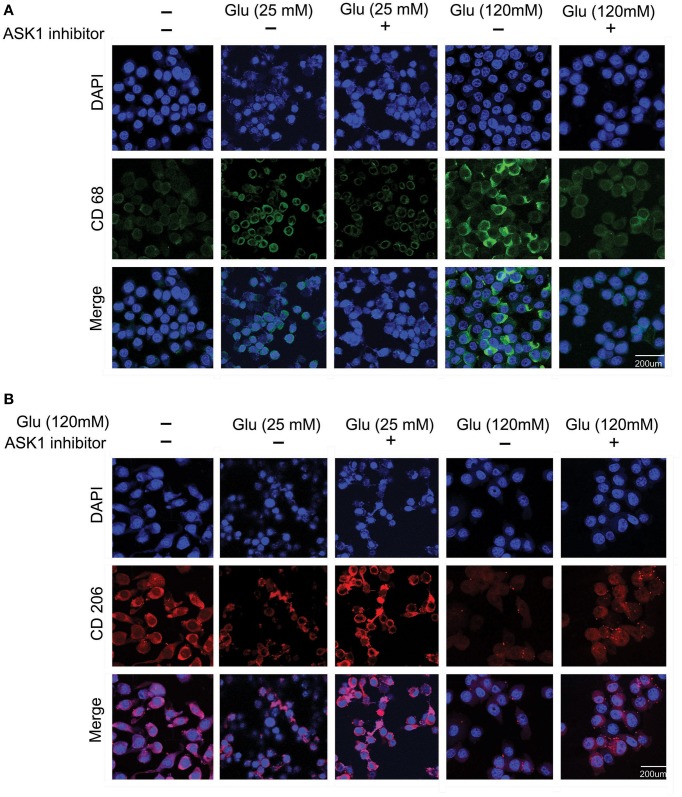
**The immunocytochemical images to determine the microglia phenotype. (A)** The expression of CD 68 (considered as the marker of M1 type microglia) was evaluated by immunocytochemistry. This image showed that high glucose 120 mM treated microglia expressed more the CD 68 protein in microglia than the untreated microglia. Also, the ASK1 inhibitor pre-treated microglia showed the attenuation of CD 68 expression compared with the normal microglia. **(B)** The expression of CD 206 (considered as the marker of M2 type microglia) was examined by immunocytochemistry. This image showed that high glucose 120 mM treated microglia expressed lesser the CD 206 protein in microglia than the untreated microglia. In addition, the ASK1 inhibitor pre-treated microglia showed the increase of CD 206 expression compared with the normal microglia. Scale bar: 200 μm, 4′, 6-diamidino-2-phenylindole (DAPI): blue, CD 68: green, CD 206: red. Glu (25 mM): glucose 25 mM treatment, Glu (120 mM): glucose 120 mM treatment.

To observe the function of microglia, we investigated the expression of CD68 (Figure 2A) and CD206 (Figure 2B) in 25 mM and 120 mM high glucose concentration using immunocytochemical analysis. The expression of CD68 was considerably increased in the high-glucose treatment group (120 mM) and was attenuated by the inhibition of ASK1 activation (Figure 2A). The expression of CD206 was considerably reduced in the high-glucose treatment group (120 mM) and was slightly increased by the inhibition of ASK1 (Figure 2B).

### Measurement of cytokine levels in microglia after high-glucose injury

**Figure 3:**
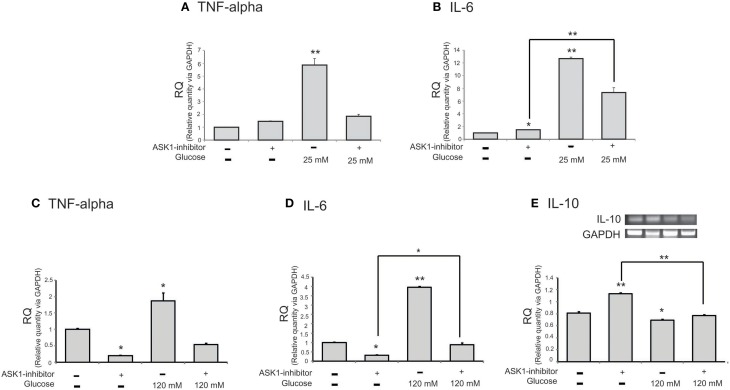
**The mRNA level of cytokines on microglia in high glucose stress**. The mRNA levels of TNF-alpha, IL-6, IL-10 were measured using quantitative real time–PCR **(A–D)** and reverse transcription-PCR **(E)**. **(A)** The mRNA level of TNF-alpha was increased in the high glucose treatment group (25 mM) and reduced in ASK1 inhibitor pre-treated high glucose group. **(B)** The mRNA amount of IL-6 was increased in the high glucose treatment group (25 mM) and significantly decreased in ASK1 inhibitor pre-treated high glucose group. **(C)** The mRNA level of TNF-alpha was increased in the high glucose treatment group (120 mM) and reduced in ASK1 inhibitor pre-treated high glucose group. **(D)** The mRNA amount of IL-6 was increased in the high glucose treatment group (120 mM) and significantly decreased in ASK1 inhibitor pre-treated high glucose group (120 mM). **(E)** The mRNA level of IL-10 exhibited decrease of expression in the high glucose treatment group (120 mM) whereas it significantly increased in ASK1 inhibitor pre-treated high glucose group (120 mM). GAPDH was used as a control. Each experiment included the 3 repeats per condition. Data were expressed as mean ± S.E.M, and were analyzed statistically using One-Way ANOVA, followed by Bonferroni's *post hoc*. ^*^p < 0.05, ^**^p < 0.001. RQ, relative quantitative value of mRNA via GAPDH.

To examine the mRNA level of cytokines in microglia following the inhibition of ASK1 activation in high-glucose injury (25 mM and 120 mM), we conducted quantitative real-time PCR (Figures 3A–D) and RT-PCR (Figure 3E). The mRNA level of pro-inflammatory cytokine TNF-α in microglia was increased in the high-glucose treatment group (Figures 3A,C) and was dramatically reduced by ASK1 inhibition (Figures 3A,C). The mRNA level of pro-inflammatory cytokine IL-6 in microglia was significantly increased in the high-glucose treatment group (Figures 3B,D) and was decreased by ASK1 inhibition (Figure 3B). The mRNA level of anti-inflammatory cytokine IL-10 in microglia was significantly decreased in the high-glucose treatment group (Figure 3E) and tended to increase after ASK1 inhibition (Figure 3E). Taken together, these results indicate that the inhibition of ASK1 activation regulates the expression of cytokines.

### Measurement of miR-Let7A level in BV2 microglia after high-glucose injury

**Figure 4:**
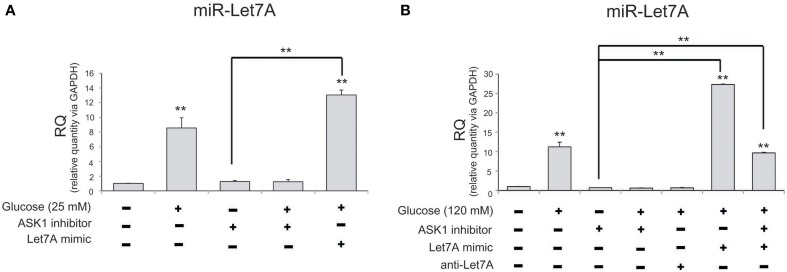
**The measurement of miR-Let7A on microglia in high glucose stress. (A)** In high glucose 25 mM treatment group, miR-Le7A level was considerably increased in comparison with the no treatment group. Under high glucose stress, ASK1 inhibitor inhibited the reduced level of miR-Let7A compared with the high glucose treatment group. The co-treatment with glucose 25 mM and miR-Let7A mimic showed higher level of miR-Le7A than high glucose treatment group. U6 was used as a control. Each experiment included the three repeats per condition. Data were expressed as mean ± S.E.M, and were analyzed statistically using One-Way ANOVA, followed by Bonferroni's *post hoc*. ^**^p < 0.001. **(B)** In high glucose 120 mM treatment group, miR-Le7A level was considerably increased compared to the no treatment group. Under high glucose stress, ASK1 inhibitor inhibited the reduced level of miR-Let7A compared with the high glucose treatment group. The co-treatment with ASK1 inhibitor and miR-Let7A mimic showed lesser level of miR-Le7A than high glucose treatment group. U6 was used as a control. Each experiment included the 3 repeats per condition. Data were expressed as mean ± S.E.M, and were analyzed statistically using One-Way ANOVA, followed by Bonferroni's *post hoc*. ^**^p < 0.001. RQ, relative quantitative value of mRNA via U6**;** ASK1 inhibitor, ASK1 inhibitor (600 nM) pre-treatment and high glucose injury group/The inhibition of ASK1 activation group; Let7A mimic, Let7A over expression group in high glucose condition; anti-Let7A, Let7A inhibition in high glucose condition; Glucose (25 mM), glucose 25 mM treatment; Glucose (120 mM), glucose 120 mM treatment.

To examine levels of miR-Let7A in microglia following the inhibition of ASK1 activation in high-glucose injury (25 mM and 120 mM), we conducted a Taqman assay (Figure 4). The level of miR-Let7A in microglia was significantly increased in the high-glucose treatment group [25 mM (Figure 4A) and 120 mM (Figure 4B)] and was reduced by ASK1 inhibition (Figure 4). The level of miR-Let7A in microglia in the high-glucose treatment group was almost the same as that in microglia with overexpression of Let7A plus ASK1 inactivation in the high-glucose condition (Figure 4).

### Relationship between ASK1 and miR-Let7A in high-glucose injury

**Figure 5:**
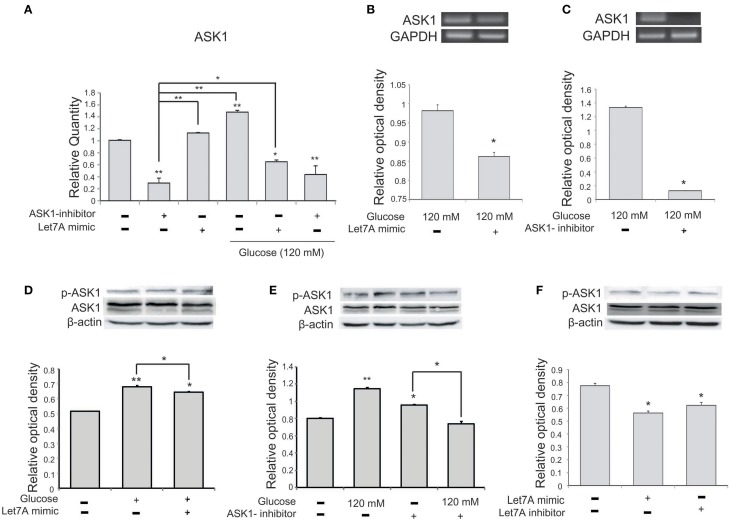
**The measurement of ASK1 on microglia in high glucose stress. (A)** The mRNA level of ASK1 was evidently increased by glucose treatment. Under high glucose stress, ASK1 inhibitor and the miR-Let7A overexpression group inhibited the decreased level of miR-Let7A compared to the normal control group. GAPDH was used as a control. Each experiment included the three repeats per condition. Data were expressed as mean ± S.E.M, and were analyzed statistically using One-Way ANOVA, followed by Bonferroni's *post hoc*. ^*^p < 0.05, ^**^p < 0.001, **(B)** The overexpression of Let7A was significantly decreased the mRNA of ASK1 in glucose 120 mM treatment condition in comparison with the only glucose treatment group. GAPDH was used as a control. Each experiment included the three repeats per condition. Data were expressed as mean ± S.E.M, and were analyzed statistically using One-Way ANOVA, followed by Bonferroni's *post hoc*. ^*^p < 0.05. **(C)** The inhibition of ASK1 was significantly attenuated the mRNA of ASK1 in glucose 120 mM treatment condition in comparison with the only glucose treatment group. GAPDH was used as a control. Each experiment included the three repeats per condition. Data were expressed as mean ± S.E.M, and were analyzed statistically using One-Way ANOVA, followed by Bonferroni's *post hoc*. ^*^p < 0.05. **(D)** The phosphorylation of ASK1/ASK1 level was increased by glucose 120 mM treatment compared to the normal control group. Also the activation of ASK1 was reduced in the glucose with Let7A overexpression in comparison with the only glucose treatment group. ß-actin was used as a control. Each experiment included the three repeats per condition. Data were expressed as mean ± S.E.M, and were analyzed statistically using One-Way ANOVA, followed by Bonferroni's *post hoc*. ^*^p < 0.05, ^**^p < 0.001. **(E)** The activation of ASK1 was reduced by ASK1 inhibitor treatment under glucose 120 mM treatment in comparison with the ASK1 inhibitor treatment group. β-actin was used as a control. Each experiment included the three repeats per condition. Data were expressed as mean ± S.E.M, and were analyzed statistically using One-Way ANOVA, followed by Bonferroni's *post hoc*. ^*^p < 0.05, ^**^p < 0.001. **(F)** The activation of ASK1 was reduced by Let7A mimic treatment compared with the no treatment group. β-actin was used as a control. Each experiment included the three repeats per condition. Data were expressed as mean ± S.E.M, and were analyzed statistically using One-Way ANOVA, followed by Bonferroni's *post hoc*. ^*^p < 0.05. ASK1 inhibitor, ASK1 inhibitor (600 nM) pre-treatment and high glucose injury group/The inhibition of ASK1 activation group; Let7A mimic, Let7A over expression group in high glucose condition; anti-Let7A, Let7A inhibition in high glucose condition; p-ASK1: the phosphorylation of ASK1.

To confirm a relationship between miR-Let7A and ASK1 activation in microglia in high-glucose injury, we conducted quantitative real-time PCR (Figure 5A) and RT PCR (Figures 5B,C) and western blot analysis (Figures 5D–F). The mRNA level of ASK1 in microglia was significantly increased in the high-glucose treatment group and was reduced in the miR-Let7A mimic transfection group (Figure 5A). Also, the mRNA level of ASK1 was reduced by Let7A mimic transfection under glucose 120 mM treatment condition (Figure 5A). The mRNA level of ASK1 in BV2 cells was reduced by Let7A mimic treatment (Figure 5B), and ASK1 inhibitor treatment (Figure 5C) in comparison with the only glucose 120 mM treatment group (Figures 5B,C). The activation of ASK1 [the phosphorylation of ASK1(p-ASK1)/ASK1 protein level] was increased in glucose 120 mM treatment group, while the increased phosphorylation of ASK1 in glucose 120 mM treatment was attenuated by the inhibition of ASK1 (Figure 5E). The activation of ASK1 was reduced in Let7A treatment group (Figure 5F).

### Effect of ASK1 inhibition and miR-Let7A overexpression on cytokine expression in high-glucose injury

**Figure 6:**
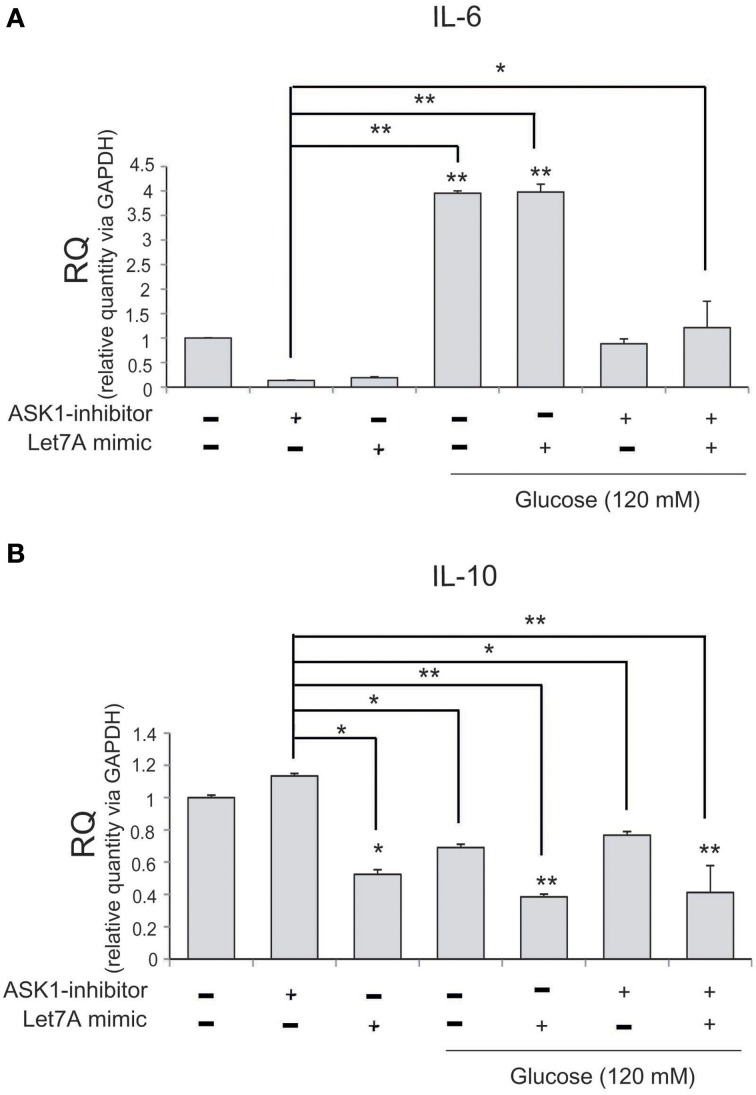
**The mRNA level of cytokines on microglia in high glucose stress**. The mRNA levels of IL-6, IL-10 in ASK1 inhibition and miR-Let7A overexpression were measured using quantitative real time–PCR. **(A)** The mRNA level of IL-6 was increased in the high glucose treatment group and reduced in ASK1 inhibitor pre-treatment group. In high glucose condition, IL-6 mRNA level was attenuated as the inhibition of ASK1 activation. **(B)** The mRNA level of IL-10 was reduced in the high glucose treatment group and increased in ASK1 inhibitor pre-treatment group. In high glucose condition, IL-10 mRNA level was attenuated through the overexpression of miR-Let7A whereas the inhibition of ASK1 activation was promoted the expression of IL-10. GAPDH was used as a control. Each experiment included the three repeats per condition. Data were expressed as mean ± S.E.M, and were analyzed statistically using One-Way ANOVA, followed by Bonferroni's *post hoc*. ^*^p < 0.05, ^**^p < 0.001. RQ, relative quantitative value of mRNA.

To examine changes in cytokine mRNA levels in microglia after ASK1 inactivation and miR-Let7A overexpression under high-glucose injury, we conducted quantitative real-time PCR. The mRNA level of pro-inflammatory cytokine IL-6 in microglia was significantly increased in the high-glucose treatment group (Figure 6A) and was also increased in the Let7A mimic transfection group. In addition, the expression of IL-6 was decreased in the ASK1 inactivation group and in the ASK1 inhibition plus Let7A overexpression group after high-glucose injury (Figure 6A). The mRNA level of anti-inflammatory cytokine IL-10 in microglia was reduced in the high-glucose treatment group (Figure 6B) and was increased in the miR-Let7A overexpression group. In addition, the expression of IL-10 was increased in the ASK1 inactivation group in the high-glucose condition compared with that in the high-glucose treatment alone group. In the inhibition of ASK1 plus Let7A overexpression in high-glucose injury group, the expression of IL-10 was slightly increased compared with that in the Let7A mimic transfection group (Figure 6B).

### Alteration of N-Myc and c-Myc expression by ASK1 inhibition and Let7A overexpression in high-glucose injury

**Figure 7:**
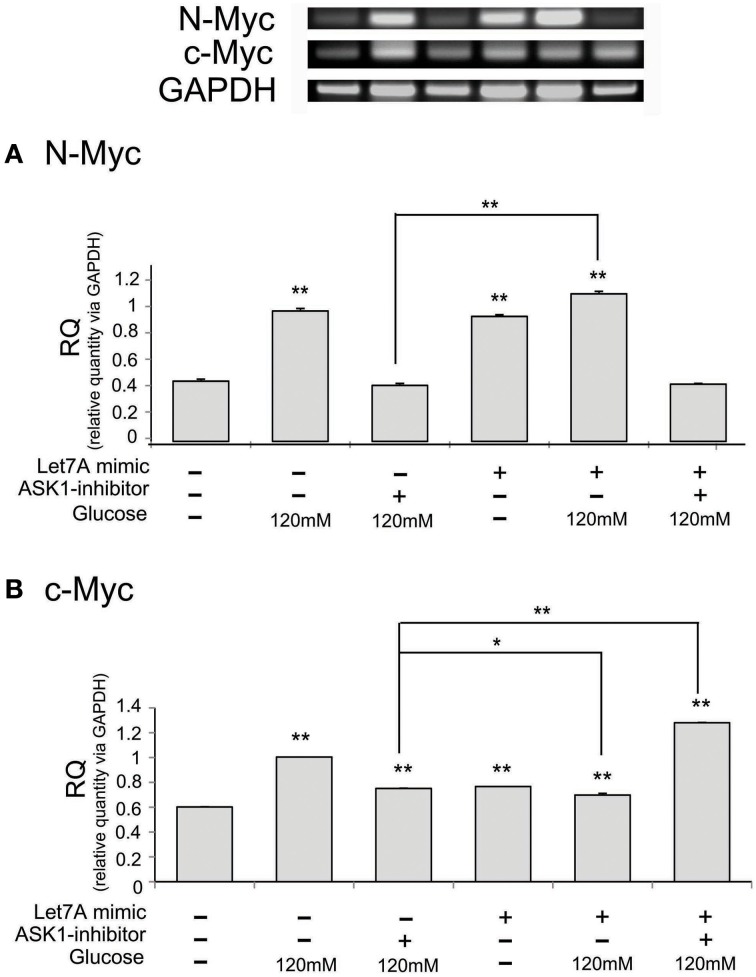
**The mRNA level of N-Myc and c-Myc on microglia in high glucose stress**. The mRNA levels of N-Myc and c-Myc were measured using reverse transcription—PCR **(A,B)**. **(A)** The mRNA level of N-Myc evidently exhibited increase of expression in the high glucose treatment group whereas it was decreased in ASK1 inhibitor pre-treated group. In ASK1 inhibition with Let7A overexpression group, microglia expressed lower N-Myc mRNA level in high glucose condition than only high glucose treatment group. **(B)** The mRNA level of c-Myc showed increase of expression in the high glucose treatment group whereas it significantly was attenuated in ASK1 inhibitor pre-treated high glucose group. In ASK1 inhibition with Let7A overexpression group, microglia was increased c-Myc mRNA level in high glucose condition than only high glucose treatment group. GAPDH was used as a control. Each experiment included the four repeats per condition. Data were expressed as mean ± S.E.M, and were analyzed statistically using One-Way ANOVA, followed by Bonferroni's *post hoc*. ^*^p < 0.05, ^**^p < 0.001. RQ, relative quantitative value of mRNA. ASK1 inhibitor, ASK1 inhibitor (600 nM) pre-treatment and high glucose injury group/The inhibition of ASK1 activation group; Let7A mimic. Let7A over expression group in high glucose condition.

To examine the regulation of N-Myc and c-Myc by ASK1 inhibition and miR-Le7A overexpression, we analyzed the mRNA level of these factors using RT-PCR (Figure 7). The mRNA level of N-Myc was increased in the high-glucose condition (Figure 7A). The inhibition of ASK1 activation markedly decreased N-Myc expression, whereas miR-Let7A overexpression did not result in large changes in N-Myc mRNA levels in the high-glucose condition (Figure 7A). Moreover, the inhibition of ASK1 in the Let7A mimic transfection condition decreased N-Myc mRNA level to almost the same value as that for ASK1 inhibition under the high-glucose condition (Figure 7A). Based on our findings, we assume that miR-Let7A links ASK1 to N-Myc in the high-glucose condition. In addition, the mRNA level of c-Myc was significantly increased in the high-glucose condition (Figure 7B). The inhibition of ASK1 activation decreased c-Myc expression, and miR-Let7A overexpression led to a slight decrease of c-Myc mRNA level under the high-glucose condition compared with that for the high-glucose treatment alone group (Figure 7B). Moreover, the inhibition of ASK1 plus Let7A overexpression increased c-Myc mRNA level in the high-glucose condition (Figure 7B).

## Discussion

In the CNS, elevated glucose levels lead to severe neuronal death and impaired cognition (Duarte et al., 2008; McCrimmon et al., 2012). In hyperglycemic conditions, ASK1 is activated, involving apoptotic gene expression (F. Wang et al., 2015). miRNAs are small, 19− to 23−nucleotide RNA molecules that play a role in regulating protein expression in cells (Cai et al., 2010; Feng et al., 2010; Lu et al., 2010). miRNAs have been implicated in various cellular processes such as proliferation and apoptosis (Bartel, 2004; Bentwich et al., 2005). In particular, previous studies suggest that miR-Let7A is associated with apoptotic signaling in high-glucose conditions (Urbich et al., 2008; Iliopoulos et al., 2009; Zhu et al., 2011; Perez et al., 2013). Our results confirm the involvement of ASK1 in microglia cell viability and activation in high-glucose conditions, consistent with findings that ASK1 is associated with high-glucose-induced endoplasmic reticulum stress and apoptosis (Jiang et al., 2015; Wang et al., 2015). In addition, our results regarding the M1-type marker CD 68 (Lemstra et al., 2007; Wojtera et al., 2012) and M2-type marker CD 206 (Stein et al., 1992; Mulder et al., 2014) indicate that the inhibition of ASK1 may induce activation of M2-type microglia and affect signaling that suppresses the inflammation response, suggesting that M2-type macrophages protect cells from inflammation (Varin and Gordon, 2009; Colton and Wilcock, 2010; Cherry et al., 2014). In the present study, a high-glucose state led to increases in the levels of pro-inflammatory cytokines such as TNF-α and IL-6 (Varnum and Ikezu, 2012) and decreases in the levels of anti-inflammatory cytokine such as IL-10 (Ledeboer et al., 2000). Furthermore, we found that inhibition of ASK1 attenuated the release of pro-inflammatory cytokines and promoted the release of anti-inflammatory cytokine such as IL-10 in high-glucose stress. Moreover, more miR-Let7A expression occurred in the ASK1 activation condition than in the normal condition. Based on our results, we assume that miR-Let7A may be linked to the activation of ASK1 and may be involved in the expression of cytokines in hyperglycemia. In the present study, the transfection of miR-Let7A and anti-miR-Let7A led to ASK1 activation and altered the expression of cytokines. In particular, miR-Let7A tended to reduce pro-inflammatory cytokines more than anti-inflammatory cytokines. miR-Let7A regulated the expression of cytokines more in the ASK1 inhibitor co-treatment group than the miR-Let7A mimic transfection only group. Several studies demonstrate that anti-inflammatory cytokines are regulated through inhibition of ASK1 activation (Liu et al., 2012), and ASK1 is involved in the production of inflammatory cytokines in macrophages (Matsuzawa et al., 2005; Osaka et al., 2007; Yuk et al., 2009). Considering previous evidence, our results indicate that Let7A miRNA may strongly regulate the expression of inflammatory cytokines by controlling ASK1 activation. Specifically, our results show that miR-Let7A may affect the expression of anti-inflammatory cytokines including IL-10 by regulating ASK1 activation under glucose-induced stress. IL-10 is an anti-inflammatory cytokine that inhibits the production of pro-inflammatory cytokines including TNF-α, IL-1, and IL-6 (Joyce et al., 1994; Joyce and Steer, 1996; Sawada et al., 1999) in response to oxidative stress (Ledeboer et al., 2000; Molina-Holgado et al., 2001; Szczepanik et al., 2001), and its expression is also correlated with protection against neuronal damage (de Waal Malefyt et al., 1991; Fiorentino et al., 1991; Froen et al., 2002). Also, gene delivery of either IL-10 in the rat hippocampus alleviates deficits in learning and memory function (Lynch et al., 2004; Nolan et al., 2005). Based on our results, the promotion of IL-10 by miR-Let7A transfection may suppress the inflammatory response and protect neurons via ASK1 activation in hyperglycemic conditions. The Myc protein, as an unstable nuclear phosphorylated protein, is involved in many cellular mechanisms (Hann and Eisenman, 1984; Ramsay et al., 1986). c-Myc induces apoptosis signaling with mitogenic signals (Prendergast, 1999). c-Myc protein is phosphorylated at both Ser-62 and Ser-71 by JNK (Noguchi et al., 1999) and is linked to ASK1 signaling (Noguchi et al., 2001). c-Myc triggers the activation of ASK1 and the mitochondrial apoptosis pathway (Evan and Littlewood, 1998; Noguchi et al., 1999; Desbiens et al., 2003) and induces cell proliferation (Sears et al., 1999). Furthermore, c-Myc is a crucial factor in both ribosome biogenesis and p53 stabilization (Menne et al., 2007; Dai and Lu, 2008; Kim et al., 2011). A recent study demonstrates that the inhibition of p38 and ASK1 reduces N-myc transcript levels (Guldal et al., 2012). N-myc triggers the apoptosis of neuroblastoma in hypoxia stress (Rossler et al., 2001). In hepatocytes, N-myc also leads to apoptosis following serum deprivation (Ueda and Ganem, 1996). N-myc was originally identified by oncogene expression profiling of human neuroblastoma cells (Schwab et al., 1983). The elevated expression of N-myc increases the susceptibility of neuroblastoma cells to diverse types of cell death signaling (Schweigerer et al., 1990; Malynn et al., 2000; Rossler et al., 2001). In addition, N-myc plays a crucial role in microglia function by regulating inflammatory mediators (Jung et al., 2005). Several researchers have discovered that the N-myc downstream-regulated gene 2 (Ndrg2) plays a key role in reactive astrogliosis involving IL-6/STAT3 signaling (Takarada-Iemata et al., 2014), and N-myc controls cytokine-induced regulator IRE1 protein and c-Jun N-terminal kinase in pancreatic beta cells (Brozzi et al., 2014). Considering our results, we speculate that miR-Let7A regulates the expression of c-Myc and N-myc by involving ASK1 activation in high-glucose conditions and that miR-Let7A may also be associated with the cytokine secretion pathway by regulating c-Myc and N-Myc protein. In conclusion, we suggest that miR-Let7A may affect microglia by involving ASK1 activation in hyperglycemia. The results of the present study increase our understanding of the relationship between miR-Let7A and ASK1 and the function of microglia in hyperglycemia-induced oxidative stress.

## Author contributions

JS performed the experiences and drafted the manuscript. JL discussed the findings and helped draft the manuscript.

### Conflict of interest statement

The authors declare that the research was conducted in the absence of any commercial or financial relationships that could be construed as a potential conflict of interest.
